# In‐hospital mortality in SARS‐CoV‐2 stratified by hemoglobin levels: A retrospective study

**DOI:** 10.1002/jha2.195

**Published:** 2021-05-05

**Authors:** Mohammed Al‐Jarallah, Rajesh Rajan, Ahmad Al Saber, Jiazhu Pan, Ahmad T. Al‐Sultan, Hassan Abdelnaby, Moudhi Alroomi, Raja Dashti, Wael Aboelhassan, Farah Almutairi, Mohammed Abdullah, Naser Alotaibi, Mohammad Al Saleh, Noor AlNasrallah, Bader Al‐Bader, Haya Malhas, Maryam Ramadhan, Mahdy Hamza, Kobalava D. Zhanna

**Affiliations:** ^1^ Department of Cardiology, Sabah Al Ahmed Cardiac Centre Al Amiri Hospital, Kuwait Kuwait; ^2^ Department of Mathematics and Statistics University of Strathclyde Glasgow UK; ^3^ Department of Community Medicine and Behavioural Sciences, College of Medicine Kuwait University Kuwait City Kuwait; ^4^ Department of Endemic and Infectious diseases, Faculty of Medicine, Suez Canal University, Ismailia, Egypt. Department of Internal Medicine, Division of gastroenterology, Al Sabah Hospital Ministry of Health, Shuwaikh Medical Area Kuwait; ^5^ Department of Infectious Diseases, Infectious Diseases Hospital Ministry of Health, Shuwaikh Medical Area Kuwait; ^6^ Department of Internal Medicine, Division of gastroenterology, Jaber Al Ahmed Hospital Ministry of Health, South Surra Kuwait; ^7^ Department of medicine, Farwaniya Hospital Ministry of Health Farwaniya Kuwait; ^8^ Department of Medicine, Al Adan Hospital Ministry of Health, Hadiya Kuwait; ^9^ Department of Emergency Medicine, Mubarak Al‐Kabeer Hospital Ministry of Health Jabriya Kuwait; ^10^ Department of Obstetric and Gynecology, Maternity Hospital Ministry of Health Sabah Area, Shuwaikh Medical Area Kuwait; ^11^ Department of Medical Imaging, Al Adan Hospital Ministry of Health, Hadiya Kuwait; ^12^ Department of Internal Medicine with the Subspecialty of Cardiology and Functional Diagnostics Named after V.S. Moiseev, Institute of Medicine Peoples’ Friendship University of Russia (RUDN University) Moscow Russian Federation

**Keywords:** anemia, COVID‐19, hemoglobin, in‐hospital mortality, SARS‐CoV‐2

## Abstract

This study is to estimate in‐hospital mortality in severe acute respiratory syndrome coronavirus 2 (SARS‐CoV‐2) patients stratified by hemoglobin (Hb) level. Patients were stratified according to hemoglobin level into two groups, that is, Hb <100 g/L and Hb >100 g/L. A total of 6931 patients were included. Of these, 6377 (92%) patients had hemoglobin levels >100 g/L. The mean age was 44 ± 17 years, and 66% of the patients were males. The median length of overall hospital stay was 13 days [2; 31]. The remaining 554 (8%) patients had a hemoglobin level <100 g/L. Overall mortality was 176 patients (2.54%) but was significantly higher in the group with hemoglobin levels <100 g/L (124, 22.4%) than in the group with hemoglobin levels >100 g/L (52, 0.82%). Risk factors associated with increased mortality were determined by multivariate analysis. The Kaplan‐Meier survival analysis showed hemoglobin as a predictor of mortality. Cox proportional hazards regression coefficients for hemoglobin for the HB ≤ 100 category of hemoglobin were significant, B = 2.79, SE = 0.17, and HR = 16.34, *p* < 0.001. Multivariate logistic regression showed Hb < 100 g/L had a higher cumulative all‐cause in‐hospital mortality (22.4% vs. 0.8%; adjusted odds ratio [aOR], 0.33; 95% [CI]: [0.20–0.55]; *p* < 0.001). In this study, hemoglobin levels <100 g/L were found to be an independent predictor of in‐hospital mortality.

## 1 INTRODUCTION

In severe acute respiratory syndrome coronavirus 2 (SARS‐CoV‐2) infection, the level of serum hemoglobin remains a predictor of adverse events. Low hemoglobin (Hb) levels in SARS‐CoV‐2 are associated with the development of respiratory failure. The requirement for mechanical ventilation was observed to be higher with low Hb levels [1]. Low hemoglobin levels in the setting of SARS‐CoV‐2 pneumonia may exacerbate O2 desaturation and are a predictor of poor prognosis [2]. Disseminated intravascular coagulation‐related low Hb levels are seen in SARS‐CoV‐2 [3]. Many studies have shown that ventilator‐dependent critically ill SARS‐CoV‐2 patients have low Hb levels [4]. Anemia is an independent predictor of severe SARS‐CoV‐2 infection as well as overall mortality [5].

## 2 METHODS

Participants and the study design comprised a total of 6931 confirmed COVID‐19 patients, both Kuwaitis and non‐Kuwaitis above the age of 18, who were enrolled in this retrospective cohort study between February 26 and September 8, 2020. The data were collected from electronic medical records from two tertiary care hospitals in Kuwait, Jaber Al‐Ahmed Hospital and Al Adan General Hospital.

SARS‐CoV‐2 infection was confirmed by a positive Reverse Transcription Polymerase Chain Reaction (RT‐PCR) swab from the nasopharynx. The treatment and care of all patients were standardized according to protocol by the Ministry of Health in Kuwait. The standing committee for coordination of health and medical research at the Ministry of Health in Kuwait approved the protocol and waived the requirement of informed consent (Institutional review board number 2020/1422). Patients were stratified according to hemoglobin levels into two groups, that is, Hb <100 g/L and Hb >100 g/L. “The intended outcome” was a COVID‐19‐related death based on ICD 10 code U07.1. The following variables were assessed: sociodemographic characteristics, underlying comorbidities, clinical presentations, laboratory results, and duration of ICU and in‐hospital stays. An electronic case‐record form (CRF) was used for data entry. The data from medical records from each participating site were submitted to an electronic CRF by qualified doctors.

## 3 STATISTICAL ANALYSIS

Descriptive statistics are used to present the data. Categorical variables are summarized as frequencies and percentages and were analyzed using Pearson's χ^2^ test. Continuous variables are summarized using the mean and standard deviation. To evaluate the impact of Hb level (Hb <100 g/L and Hb >100 g/L) on all‐cause mortality, we used multivariable logistic regression. The odds ratios (ORs) for in‐hospital all‐cause mortality status were adjusted for sex, age, white blood cells, platelet counts, neutrophils, and hemoglobin levels.

A Cox proportional hazards model was performed to determine whether hemoglobin had a significant effect on the hazard of mortality. The live category of mortality was used to indicate survival while the dead category was used to represent a hazard event. The level of significance was set at *p* < 0.05 a priori. Statistical analyses were conducted using R statistical packages [6] and SPSS version 27 (SPSS, Chicago, IL, USA).

## 4 RESULTS

A total of 6931 SARS‐CoV‐2‐positive patients were included in the study; the mean patient age was 44 ± 17 years, and 66% of the patients were male. We observed that 6377 (92%) of the patients had hemoglobin levels >100 g/L. The median length of overall hospital stay was 13 days [2; 31]. In this study, 554 (8%) patients had a hemoglobin level <100 g/L. The overall mortality was 176 patients (2.54%), and the mortality rate was higher in the group with hemoglobin levels <100 g/L (124, 22.4%) than in the group with hemoglobin levels >100 g/L (52, 0.82%). Risk factors associated with increased mortality were established by multivariate logistic regression. The median duration of hospitalization was 13.0 (2.00, 31.0) days. The average length of hospital stay was higher in the group with lower levels of hemoglobin (<100 g/L), 16.5 (2.00, 39.5) days, while in the group with higher levels of hemoglobin (greater than or equal to 100 g/L), this figure was 13.0 (2.00, 29.0) days (*p* < 0.001). The overall cumulative all‐cause in‐hospital mortality was 2.54% (*n* = 176). The mortality rate was higher in the group with lower hemoglobin levels (124, 22.4%) than in the group with higher hemoglobin levels 100 g/L (52, 0.82%), (*p* < 0.001) (Table 1). Individuals with low hemoglobin levels < 100 g/L had a higher cumulative all‐cause in‐hospital mortality than those with high hemoglobin levels > 100 g/L (22.4% vs. 0.8%; adjusted OR (aOR), 0.33; 95% confidence interval (CI): [0.20–0.55]; *p* < 0.001). Age had no significant impact among the groups with regard to the all‐cause cumulative in‐hospital mortality (*p* = 0.809). Male sex had a significant impact on cumulative all‐cause in‐hospital mortality (6.8% vs. 2.3%; aOR, 2.63; 95% confidence interval (CI): [1.49, 4.80]; *p* < 0.001) (Table 2).

**TABLE 1:** Demographics and clinical characteristics of the cohort stratified by hemoglobin (HB) levels among patients admitted with SARS‐CoV 2

Demographics and clinical characteristics	[ALL] *N = 6931*	HB < = 100 *N = 554*	HB > 100 *N = 6377*	*p*‐value	N
Age, mean (SD), years	44.1 ± 17.2	55.1 ± 14.6	42.6 ± 16.9	<0.001	3360
Male, n (%)	2221 (66.1%)	239 (61.0%)	1982 (66.8%)	0.026	3360
ICU admission (days), IQR	0.00 (0.00; 4.00)	1.00 (0.00; 12.0)	0.00 (0.00; 3.00)	<0.001	3360
Admission to discharge (number of days in hospital), IQR	13.0 (2.00; 31.0)	16.5 (2.00; 39.5)	13.0 (2.00; 29.0)	<0.001	2900
ICU to discharge (days), IQR	9.00 (0.00; 38.6)	8.00 (0.00; 39.0)	9.00 (0.00; 35.8)	0.459	416
Mortality, *n* (%)	176 (2.54%)	124 (22.4%)	52 (0.82%)	<0.001	6931

Abbreviations: IQR, interquartile range; SD, standard deviation.

Percentages might not add up to 100% due to rounding off.

**TABLE 2:** Multivariate logistic regression analysis of in‐hospital death in the overall study cohort

In‐hospital mortality		Alive	Dead	Univariate ^a^OR (95% CI, ^a^ *p*‐value)	Multivariate logistic regression ^a^OR (95% CI, ^a^ *p*‐value)
Gender	Female	1113 (97.7%)	26 (2.3%)	–	–
	Male	2071 (93.2%)	150 (6.8%)	3.10 (2.07‐4.83, *p* < 0.001)	2.63 (1.49‐4.80, *p* = 0.001)
Age	Mean (SD)	43.4 ± 17.1	56.6 ± 12.1	1.05 (1.04‐1.06, *p* < 0.001)	1.00 (0.99‐1.02, *p* = 0.809)
WBC	Mean (SD)	7.4 ± 3.2	18.2 ± 10.0	1.28 (1.25‐1.32, *p* < 0.001)	1.08 (1.04‐1.11, *p* < 0.001)
NEU	Mean (SD)	55.2 ± 13.6	86.8 ± 8.4	1.23 (1.20‐1.26, *p* < 0.001)	1.14 (1.11‐1.17, *p* < 0.001)
HB	HB < = 100	430 (77.6%)	124 (22.4%)	–	–
	HB > 100	6325 (99.2%)	52 (0.8%)	0.03 (0.02‐0.04, *p* < 0.001)	0.33 (0.20‐0.55, *p* < 0.001)
PLT	Mean (SD)	288.3 ± 102.5	217.2 ± 151.1	0.99 (0.99‐0.99, *p* < 0.001)	1.00 (0.99‐1.00, *p* < 0.001)

Number in data frame = 6931, number in model = 3344, missing = 3587, AIC = 534.7, C‐statistic = 0.982, H&L = chi‐sq(8) 6.30 (*p* = 0.614).

Abbreviation: CI, confidence interval.

^a^ OR, adjusted odds ratio; ^a^ *P*‐value, adjusted *p*‐value.

Multivariable analyses were conducted using logistic regression models utilizing the simultaneous method. The models were adjusted for gender, age, WBC (white blood cells); NEU (neutrophils); HB (heamoglobin); PLT (platelet count).

Percents are row percentages.

**FIGURE 1:**
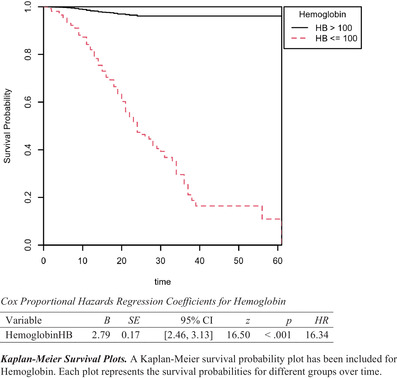
Kaplan‐Meier survival plot of mortality grouped by hemoglobin

The Kaplan‐Meier survival probability plot shows that lower hemoglobin levels were associated with higher mortality levels. The results of the model were significant based on an alpha of 0.05, LL = 295.50, and df = 1, *p* < 0.001, indicating that hemoglobin was able to predict the hazard of mortality adequately. Kaplan‐Meier survival probability plots are included for hemoglobin. Each plot represents the survival probabilities for different groups over time. Cox proportional hazards regression coefficients for hemoglobin for the HB ≤ 100 category of hemoglobin were significant, B = 2.79, SE = 0.17, and HR = 16.34, *p* < 0.001, indicating that at any particular time, an observation in the HB ≤ 100 category will have a hazard that is 16.34 times as high as the HB > 100 category. The event *B* is HB <100 g/dL (Figure 1).

## 5 DISCUSSION

The observations from this multicenter observational study showed that SARS‐CoV‐2 patients with lower hemoglobin levels are at higher risk of in‐hospital mortality than those with higher levels of >100 g/L. In this study, lower hemoglobin levels were found to be an independent predictor of in‐hospital mortality. Male sex had a significant impact on cumulative all‐cause in‐hospital mortality among the group with low hemoglobin levels. Among the two groups, age showed no significant impact on all‐cause cumulative in‐hospital mortality.

The incidence of anemia is reported in all types of pneumonia and ranges between 7% and 15% [7, 8, 9]. In SARS and SARS‐CoV‐2, the incidence of anemia ranges between 16% and 53% [5, 10]. Lower hemoglobin levels can lead to a decrease in tissue oxygen delivery, as the Hb concentration is interrelated with arterial oxygen content [11, 12]. SARS‐CoV‐2‐related infection may also alter iron metabolism and reduced iron availability [13]. Virus‐induced intestinal mucosal erosion in SARS‐CoV‐2 and associated bleeding were reported [14, 15]. Hospitalized elderly SARS‐CoV‐2 patients were found to have lower levels of hemoglobin [16]. Additionally, the incidence of anemia was found to be markedly higher in critically ill SARS‐CoV‐2 patients admitted to the intensive care unit [17].

Anemia in SARS‐CoV‐2 infection may be related to cytokine‐induced inhibition of erythropoietin formation and can lead to an increased requirement for mechanical ventilation in critically ill patients [18, 19]. A systematic review of 63 studies showed that severe SARS‐CoV‐2 infection is associated with lower hemoglobin levels [20]. The association of anemia with respiratory diseases was previously proven to be a predictor of poor outcome and increased mortality [8, 21, 22]. A study by Fan et al showed that 1.6% of SARS‐CoV‐2 patients admitted to the intensive care unit underwent blood transfusion for anemia correction [23]. The majority of the studies proved anemia as an independent predictor of mortality [24, 25].

Our study was focused on mortality, and therefore we did not report other outcome variables. We did not use the cutoff values as defined for anemia. Mild, moderate, and severe anemia classifications were not followed. There were many missing values when analyzing the data.

**In conclusion** of our study, SARS‐CoV‐2 patients with hemoglobin <100 g/L have a 67% higher risk of in‐hospital mortality than those with hemoglobin >100 g/L. In this study, hemoglobin levels <100 g/L were found to be an independent predictor of in‐hospital mortality. The length of hospital stay was longer with hemoglobin <100 g/L. Male sex had a significant impact on cumulative all‐cause in‐hospital mortality.

## AKNOWLEDGMENTS

Authors would like to thank Prof. Dr. Peter A Brady.

## CONFLICT OF INTEREST

No conflict of interest exists for any author on this manuscript.

## AUTHOR CONTRIBUTIONS

Mohammed Al‐Jarallah participated in analysis and manuscript preparation. Rajesh Rajan participated in data analysis and manuscript preparation. Ahmad Al Saber and Jiazhu Pan did the statistical analysis as well as manuscript review. All authors had access to data and take responsibility for the integrity of data and the accuracy of data analysis. All authors have read and approved the manuscript.

## ETHICS APPROVAL STATEMENT

This study was approved by the ethics committee and Ministry of Health Kuwait.

## PATIENT CONSENT STATEMENT

Patient consented was not mandated for this retrospective observational study. Permission to reproduce material from other sources: No material from other sources is included in this study.

## CLINICAL TRIAL REGISTRATION

This study was not a clinical trial.

## NOVELTY STATEMENT

This study mainly focused on the clinical significance of hemoglobin levels while treating SAR‐CoV‐2 infection. These results will help the clinicians to categorize the patients as the low hemoglobin levels were proven to be an independent predictor in‐hospital mortality. This will help the clinicians for early initiation of critical care strategies for such patients and thereby may reduce mortality and related complications to an extent.

## Data Availability

The data that support the findings of this study are available on request from the corresponding author. The data are not publicly available due to privacy or ethical restrictions.
